# Zika Virus, Elevation, and Transmission Risk

**DOI:** 10.1371/currents.outbreaks.a832cf06c4bf89fb2e15cb29d374f9de

**Published:** 2016-05-09

**Authors:** A. Townsend Peterson, Jorge Osorio, Huijie Qiao, Luis E. Escobar

**Affiliations:** Biodiversity Institute, University of Kansas, Lawrence, Kansas, USA; Department of Pathobiological Sciences, University of Wisconsin-Madison, Madison, Wisconsin, USA; Key Laboratory of Animal Ecology and Conservation Biology, Institute of Zoology, Chinese Academy of Sciences, Beijing, China; Minnesota Aquatic Invasive Species Research Center and Department of Veterinary Population Medicine, University of Minnesota, Saint Paul, Minnesota, USA

**Keywords:** Aedes, climate, disease outbreak, travel advisory, Zika

## Abstract

Introduction: Zika virus has appeared in the Americas in the form of a major outbreak, and is now known to cause birth defects when pregnant women are infected. As a result, the Centers for Disease Control and Prevention issued travel guidelines, in the form of an elevational risk definition: destinations below 2000m are considered as at-risk.

Methods: We explored the distribution of known Zika virus vector mosquito species in relation to climatic conditions, elevation, latitude, and air traffic connections to the United States.

Results: In view of the tropical and subtropical nature of the mosquito species that are the primary Zika virus vectors, we point out that climate varies rather dramatically with respect to elevation and latitude, such that a single elevational criterion will be a poor predictor of potential for transmission.

Discussion: We suggest an initial adjustment would consider latitude in addition to elevation; a more definitive, quantitative analysis of risk would consider variables of ecology, climate, human condition, and connectivity of areas.

## Article

Zika virus is a flavivirus that is transmitted by aedine mosquito vectors, and that is showing a clear trend towards “emergence” in recent years^1^. The Centers for Disease Control and Prevention (CDC) recently (11 March 2016) issued guidelines for travel by pregnant women to Latin America, in light of the burgeoning Zika virus outbreak there^2^. The new guidelines advised a cut-off for risk, based strictly on elevation, with destinations below 2000 m considered as high-risk, and those above 2000 m considered as low-risk. In the Americas, among capitol cities, this recommendation would place only Mexico City, Quito (Ecuador), Bogotá (Colombia), and La Paz (Bolivia) in the low-risk category, and all others as high-risk.

However, we believe that simplicity in this case may come at the expense of clarity and good planning. As early as 1889, the concept of life zones had been proposed, which emphasizes the point that latitude mediates elevational trends^3^: elevations that are alpine at temperate latitudes will be forested and tropical at lower latitudes. Although, as CDC pointed out^2^, most occurrences of the primary vector—*Aedes aegypti*—are from below 2000 m, in a recent compilation, occurrences of the species above 2000 m were documented from Venezuela, Peru, Colombia, and Mexico^4^. A further concern is that ample evidence indicates that Zika virus can be transmitted by additional mosquito species beyond *Ae. aegypti*, with records from the previous range of the virus^5^^,^ 6 ^,^ 7, and emerging evidence of broader potential vector distribution in the Americas^8^; this revised view of Zika virus vector distribution would broaden the elevational (and geographic) range of potential transmission considerably.

**Relationship between elevation, latitude, and minimum temperature across the Americas.**
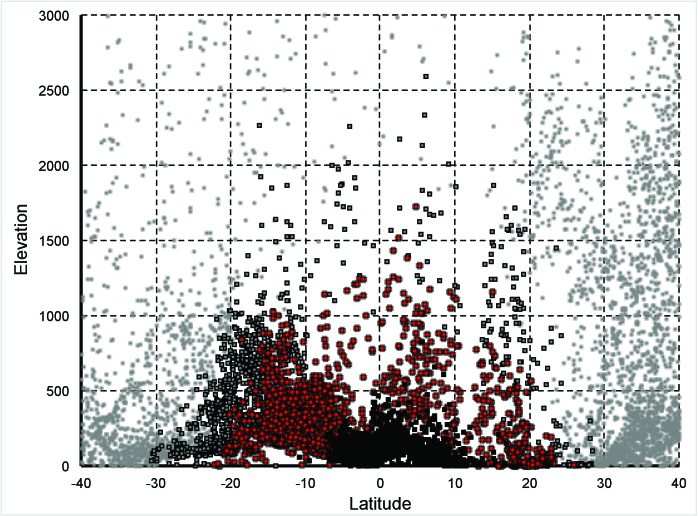
Relationship between minimum temperature of the coldest month of the year^9^, elevation, and latitude. Black squares have a minimum temperature >20°; red squares are 15-20°; gray squares with a black outline are 10-15°, and light-gray squares are <10°.

Clearly, development of simple and clear guidelines for travel advice regarding Zika virus is desirable. However, as the CDC report acknowledged^2^, causal factors will be related to temperature and other environmental dimensions, and temperatures do not obey simple rules of elevational cutoffs—rather, elevation interacts with latitude in rather dramatic ways (see Figure 1), and even then many other factors act as one translates from macroclimate to microclimate, which is the factor of importance to populations of species^10^. Certainly, then, a simple elevational cutoff that applies equally in Bogotá and in Mexico City is not supported ecologically.

High-elevation travel destinations in the Americas are relatively few in number, with some (e.g., Quito and Bogotá) being equatorial, and others at higher latitude (e.g., La Paz). Hence, we believe that a more nuanced risk mapping that takes associated environmental variation into account would be markedly more effective. As commented by CDC^2^, more complex risk mapping efforts are possible, and preliminary maps have been published for Zika in the Americas^11^^,^ 12; these risk-mapping efforts should be extended and made more complete. In the short term, an improvement over a fixed-elevation criterion is to plot air destinations for US travelers in this latitude-elevation space (Figure 2): tropical-climate destinations can be identified rather easily, at least in a macrogeographic sense.

**Elevation-latitude relationships for destination airports across the Americas.**
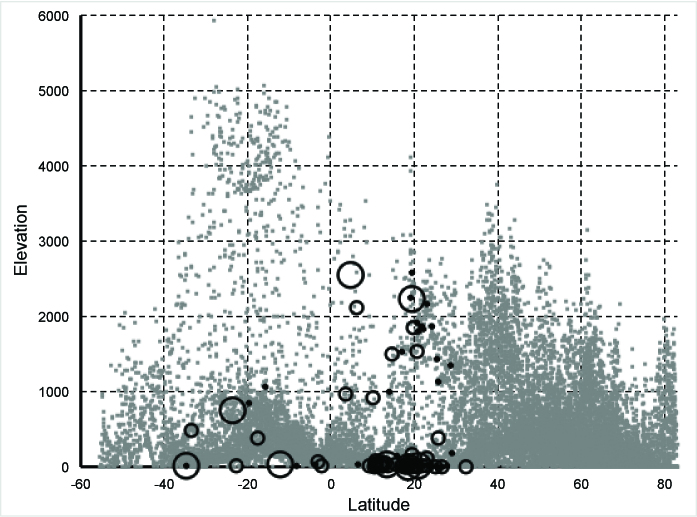
Destination airports for US travelers across the Americas (excluding the US and Canada, as Zika transmission is nil there, at least as of yet), derived from a recent risk-mapping effort focused on chikungunya virus in the Americas^13^, and plotted in an elevation-latitude space (see Figure 1). The largest circles indicate destinations with >100,000 passengers yearly; medium-sized circles 10,000-99,999 passengers yearly, and small circles <10,000 passengers yearly; gray squares indicate availability of conditions across the Americas (same data as shown in Figure 1).

**Relationships between elevation, latitude, and minimum temperature for destination airports across the Americas.**
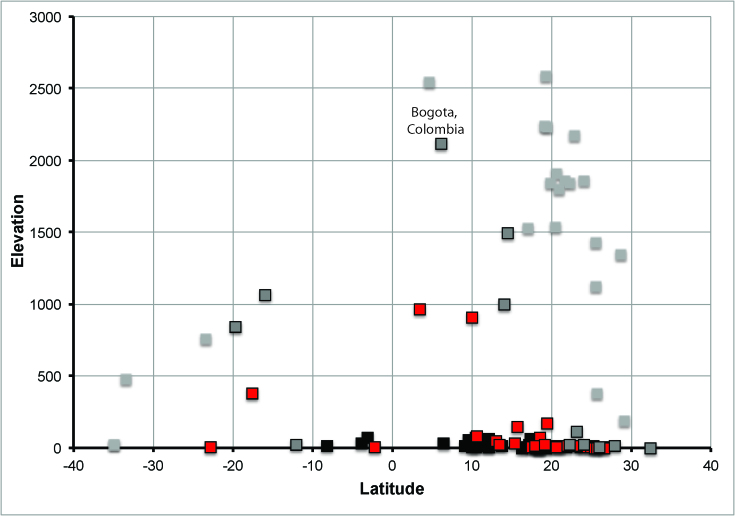
Relationship between minimum temperature of the coldest month of the year^9^, elevation, and latitude for destination airports across the Americas. Black squares have a minimum temperature >20°; red squares are 15-20°; gray squares with a black outline are 10-15°, and light-gray squares are <10°. Note the Bogotá, Colombia, airport, with an elevation above 2000 m and subtropical conditions.

We present this exploration not as a final analysis or set of recommendations, but rather as a suggestion to reconsider the too simple “elevational cutoff,” as it neglects the complex phenomena that drive disease transmission. The real-world translation of this concern is that travel to high-elevation *Equatorial* destinations is not at all without risk of Zika transmission, such that 2000 m is too low for those regions, whereas 2000 m will be too high for higher-latitude regions. At a minimum, travel guidelines should take into account the interaction between elevation and latitude; detailed climate data, suitability for key mosquito species, and information on mitigation efforts should be used to assemble a still-more-realistic approach for identifying risk areas for Zika virus transmission.

## COMPETING INTERESTS

The authors have declared that no competing interests exist.

## DATA AVAILABILITY STATEMENT

All data underlying the analyses presented in this publication are available at http://hdl.handle.net/1808/20727.
